# THE ASSOCIATION OF OBJECTIVE FINANCIAL DECISION MAKING WITH FINANCIAL LITERACY, EDUCATION, AND MATH SKILLS

**DOI:** 10.1093/geroni/igz038.2424

**Published:** 2019-11-08

**Authors:** Preeti Sunderaraman, Sarah Ho, Yaakov Stern, Stephanie Cosentino

**Affiliations:** Columbia University Medical Center, New York City, New York, United States

## Abstract

Rationale: Financial decision-making (FDM) is a critical ability with implications across the adult life-span. In healthy adults, demographic and cognitive factors impact FDM. However, the impact of these factors on FDM has yet to be fully investigated. The aim of the current study was to understand the extent to which demographics (age, education, sex), financial literacy (crystallized ability), and mathematical ability (fluid ability) influence FDM. Participants and Methods: The sample, recruited from a larger ongoing study, consisted of 73 adults; mean age=61.31 (13.76) years, mean education=15.68 (2.61) years, 59.5% female, 58% Caucasian. FDM was measured using the Financial Competence Assessment Inventory, financial literacy using a standard set of 23 questions, and math skills using WAIS-III Arithmetic, WRAT-IV Math and Cognitive Reflection Test. Results: Only variables that were significantly associated with FDM in bivariate analysis were selected for the multiple regression analysis. After adjusting for multicollinearity, stepwise multiple regression analyses revealed that the overall model with 3 predictors (education, financial literacy, WAIS-III Arithmetic) was significant (F =23.64, p < .001) and explained 50.7% of the variance in FDM. Education and WAIS-III Arithmetic predicted FDM to a higher extent than financial literacy. Conclusions: The finding that education and fluid ability has a relatively higher impact on FDM as compared to crystallized ability is important. As one ages, fluid abilities decline more rapidly than crystallized abilities. This may be one explanation for why FDM ability worsens with age. To increase confidence in these findings, future research should test these models using age-stratified analyses.

